# Health Equity for Abenaki Indigenous People: Improving Access to Quality Mental Health and Substance Use Services

**DOI:** 10.1089/heq.2022.0091

**Published:** 2022-10-06

**Authors:** Maria Mercedes Avila, Christine Begay Vining, Joshuaa Allison-Burbank, Christine Velez

**Affiliations:** ^1^ Department of Pediatrics, University of Vermont Larner College of Medicine, Burlington, Vermont, USA.; ^2^ Department of Audiology & Speech-Language Pathology, Arizona School of Health Sciences, A.T. Still University, Mesa, Arizona, USA.; ^3^ Center for Indigenous Health, Department of International Health, Johns Hopkins Bloomberg School of Public Health, Baltimore, Maryland, USA.; ^4^ Department of Social Work, University of Vermont, Burlington, Vermont, USA.

**Keywords:** Abenaki, health equity, substance use, mental health

## Abstract

**Background and Purpose:**

The purpose of this study was to learn about the Abenaki Indigenous communities' access to services, specifically, their beliefs and knowledge about different types of mental health and substance abuse services and supports within their communities.

**Methods:**

This was an exploratory qualitative study using a focus group approach. Two focus groups were conducted in spring 2020 with community members and tribal leaders from different Abenaki Bands in Franklin, Chittenden, and Addison Counties and the Northeast Kingdom in Vermont. Participants were recruited via a mix of convenience and snowball sampling approaches.

**Results:**

A total of 15 Abenaki community members participated in 2 separate focus groups, including 5 current and former Chiefs who served or were currently serving as the primary leadership of this state-recognized tribe. Members of the Abenaki community communicated that the loss and erasure of their culture combined with lack of trust of community support agencies impacted the ways in which the Abenaki community conceptualizes health and wellness for themselves and thus impacts parenting and youth substance use as well as opioid use and prescription drug misuse for participants in this study.

**Conclusion:**

Abenaki Indigenous experience many obstacles to effective prevention and intervention services. Recruitment of American Indian and Alaska Native (AI/AN) individuals, specifically Abenaki, into the health and mental health workforce can support health equity efforts for this population. Finally, better efforts to foster and support AI/AN culture, specifically Abenaki culture, can support substance use and suicide prevention with this vulnerable community.

## Background and Purpose

American Indian and Alaska Native (AI/AN) populations make up about 1.7% of the total U.S. population.^1^ While AI/AN account for only a small part of the population, they have considerably higher rates of substance use compared with other racial and ethnic groups in the United States.^1–5^ Researchers have established that legacies of colonialism and genocide are factors of historical trauma that exacerbate substance use challenges for AI/AN populations.^1,5–7^ Additional established risk factors include exposure to violence, high rates of unemployment, discrimination and racism, lack of health insurance, as well as inadequate educational access as social determinants of health contributing to higher rates of substance use for AI/AN.^1,8^ These factors combined with high rates of mental health challenges and chronic illness suggest that addressing health disparities in AI/AN should be an urgent public health issue.

Recent findings from the 2018 National Survey on Drug Use and Health (NSDUH) report that 10% of AI/AN have a substance use disorder (SUD), 4% have an illicit drug use disorder, and 7.1% have an alcohol use disorder.^8^ Substance use and mental health issues are of particular concerns among AI/AN youth, as 2018 NSDUH survey results revealed that 1 in 5 AI/ ANyoung adult (aged 18–25 years) has a substance use disorder, including 11% with an illicit drug use disorder and 10% with an alcohol use disorders.^8,9^ The Substance Abuse and Mental Health Services Administration (SAMHSA) reports that 13% of the AI/AN population in the United States need SUD treatment, but only 3.5% actually access treatment.^8^

Historical trauma, genocide and colonial legacies, and current-day forms of structural racism and violence continue to impact the health and well-being of AI/AN populations^1,5–7^ and are likely connected to high rates of substance use and mental health challenges. Cultural erasure, the forced removal of AI/AN children from their families into boarding schools, eugenics, and forced sterilization, are some of the atrocities that AI/AN have faced.^1,5–9^ The enduring trauma associated with historical and present-day racism and discrimination continues to impact the health and well-being of AI/AN populations.^9^

This qualitative exploratory study with the Abenaki people of Vermont is the only study we are aware of which examines health equity, mental health care, and substance use issues within this particular AI/AN community. The geographical area known as Vermont, New Hampshire, parts of New England, and Southern Canada are known as part of N'Dakinna, the large traditional homeland of the Abenaki people.^10^ The Abenaki do not have state recognition and are not federally recognized as a tribal nation by the U.S. government. In Vermont, violent colonial legacies of eugenics and forced sterilizations of Native women have effectively wiped-out substantial portions of this population.^11^ There is little to no peer reviewed published literature about the Abenaki, and we found no publications for this review that explicitly address mental health, substance use, health or well-being within Abenaki communities.

## Methods

This exploratory qualitative study consisted of 2 separate focus groups with a total of 15 adult Abenaki leaders and community members during spring 2020. Community members, leaders, and Chiefs from several of the Abenaki Bands, representing four different counties in Vermont, participated and were recruited via a mix of convenience and snowball sampling approaches and selected into one of the two focus groups based on convenience and availability. Both focus groups had a similar representation of chiefs or former chiefs (30%), other community leaders (30%), and community members (40%). A trusted Abenaki leader led the recruitment of participants and oral consent processes. All participants who were approached for this study chose to participate and were each paid $50.

Due to COVID-19 restrictions, focus groups took place via online videoconference, and few technical challenges were present. Focus groups members appeared to get along well, were at ease with the technological format, and were eager to partake in these discussions to share their perspectives. One of the authors is a well-known and well-respected public health equity scholar and advocate in Vermont known to many within the Abenaki community, which likely encouraged a sense of trust and safety with participants. Since the Abenaki community in Vermont is small, many participants knew each other, and thus, all engaged respectfully with one another, and the profiles of the participants (i.e., community members vs. chiefs) did not influence the information shared.

Participants, regardless of profile or group membership, shared common views and perceptions about the topics presented and the disparities and inequities that the Abenaki native people continue to experience, as these have been of concern for many years in the state and within tribal groups. Abenaki leaders (chiefs and former chiefs) had more extensive historical knowledge of disparities that helped validate the younger participants' experiences in today's time. Focus groups lasted about an hour and a half in duration, were audio recorded with participant consent, and conducted in English. A semi-structured interview guide that asked about access to services, wellness and well-being, and cultural and linguistic competence when seeking services was utilized for both focus groups.

Focus groups took place in the evenings and assembled based on availability of participants. All data were de-identified to protect the confidentiality and anonymity of participants. An inductive approach informed by Miles and Huberman guided this thematic analysis.^12^ All authors individually coded each transcript, and then, the team met several times to discuss codes, create a codebook, and to come to a consensus on theme selection. For logistical purposes, the authors were unable to member check the data. This project was approved by the University of Vermont Institutional Review Board (IRB), and Abenaki tribes and the Vermont Council on Native American Affairs were consulted to obtain appropriate consent for this study. The Abenaki people, to this date, do not have a formal IRB consent process in place for researchers to follow.

## Results

**FIG. 1.**
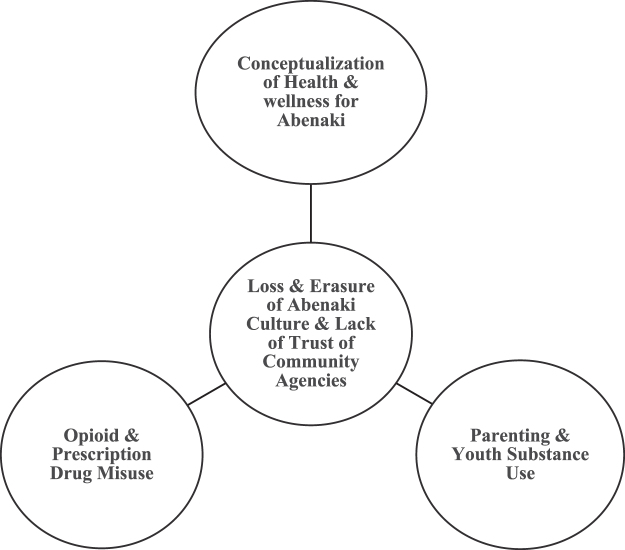
Themes of health equity for Abenaki communities in Vermont.

The following five themes emerged, which highlight the Abenaki communities' experiences with culture loss and colonialism, as well as their attitudes and beliefs about different types of mental health and substance use services and supports available, including participant reflections on wellness and well-being. These themes represent quotations from all 15 participants at least once. In our analysis, we found that the first two themes of loss and erasure of Abenaki culture, combined with lack of trust of formal supports impacted the later three themes of conceptualizing health and wellness for Abenaki communities, challenges expressed with parenting and youth substance use, and opioid and prescription drug misuse within the community represented by this sample (Fig. 1).

### Loss and erasure of Abenaki culture

Participants spoke at length about pain and trauma of the loss and erasure of Abenaki culture, as one person shared that,

If you've lost your language, you're not a functioning culture … if you have to relearn it now, that means your culture is dead. That's a settler genocidal model.

Abenaki community members shared about their attempts to reintroduce Abenaki cultural studies to local universities, as one participant noted,

“I worked for the Department of Mental Health and in … it said how people are experiencing right now loss of their community, their social circles, their traditions … that sounds so familiar … imagine … the indigenous people when … we were losing all of that in our own country, knowing it's never coming back.”

Participants shared that they have little faith that health care institutions will have the cultural competence needed to properly address their concerns, “If I could wish for one thing out of this meeting, it might be for cultural competency … in drug counseling or alcohol counseling.” Another participant shared that the history of historical trauma is clear and present for many Abenaki,

My mom … she's French-Canadian Abenaki … elderly and disabled … first, eugenics … by law … a lot of doctors … struggle with trying to communicate with her because they don't know the source of her fear.

### Lack of trust of community support agencies

Participants shared that trust of mental health providers is a major area of contention for the Abenaki community, given their recent history with medical, social, and governmental institutions:

we're trying to figure out a way to create trust in this short window of time and all of these people … had … hundreds of years of evidence to the contrary.

There is a lack of cultural competency within institutions that serve the Abenaki community:

They're always looking to have Abenaki people as someone who … used to be here.

Abenaki community members do not feel that organizations have the knowledge or skills to properly address how historical trauma drastically affects the behaviors within the Abenaki community:

I had a new family member who was sterilized … my parents' generation, that stigma stayed with them.

There is an internalized mistrust for government organizations, even by members of the Abenaki community who have worked within judiciary institutions:

I'm a former county judge, and I'm telling you, the law does not stop people from making their own personal decisions. I've got people in front of me … selling marijuana to make their child support payment.

An ex-law enforcement officer stated:

you are a mandatory ticket writer … I don't think every kid who drinks a beer has a problem. I don't necessarily know that every person that smoked marijuana has a problem. But I know they're not going to seek help from law enforcement. If the first thing you do is give them a summons that takes them into court … sometimes it's very hurtful for the family to impose these regulations.

The community has serious doubts in the effectiveness of social and legal systems' ability to rehabilitate substance abusers or legal offenders:

law enforcement's first duty in this situation is to make an arrest. Social workers first duty in the situation is to secure your children … So, it's hard to reach out for help when you have fears of more negative engagement.

### Conceptualizing health and wellness within the Abenaki community

Focus group participants explained that cultural identity and personal identity were linked to mental health:

I think of your culture as part of your wellness … if you're living in a situation where you feel supported, you feel like you can have access to food you need. You are part of a community … You're healthy. You are happy.

Furthermore, the loss of cultural and personal identity can contribute to increased substance abuse:

It's like we're all missing like a huge part of ourselves … I couldn't figure out how to access my culture for quite a while … Some of that pain is cellular … it's … generational and ancestral.

Some participants shared that Abenaki people are often asked to prove their affiliation with the tribe and thus prove their cultural identity. Abenaki people consider individuals with any percentage of Abenaki ancestry to be fully eligible to be a part of the tribe, despite the fact that the U.S. government does not recognize this tribal nation as a sovereign entity. Participants also shared a fear of repercussions related to racism and discrimination should they choose to reclaim their Abenaki identity, as one person shared:

I've known people who have lost jobs, including myself, once they found out that they were native … if I can … take advantage of white privilege, why would I want to detriment my kids[?]

Participants also shared that providers doing the work of learning about Indigenous wellness, specifically from the point of view of the Abenaki, could help improve health and well-being, as one person noted,

There are very distinct Abenaki traditions and there are layers of those … if people would be open to the possibility of understanding an indigenous perspective … the old teachings … and then trying to figure out how to bring those forward … The way it's structured right now is not really able to … learn to think about wellness from a non-Euro-American viewpoint.

### Challenges related to parenting and addressing youth substance use

A mistrust in medical and social institutions creates a barrier to parenting and to addressing substance abuse issues for both parents and their children. There is a fear that organizations that are supposed to help will fracture the family structure:

I'm dealing with someone right now that DCF [Department of Children and Families] got involved and they came to me to help them because … they don't want to lose their kids.

Such mistrust and fear in these systems dissuade persons from the Abenaki community from accessing services:

The Abenaki community has a lack of trust, to medical professionals … the best way to disseminate the information is probably through the tribal councils or leadership because they have a level of trust with their people to some degree.

Another factor that is exacerbating the issue is the constant drug reform laws that affect each generation differently:

Some of the barriers that I've personally seen are generation gaps … It's OK to smoke marijuana in Vermont because I think a lot of the kids are seeing their parents doing it now.

However, some adults feel that the most important way to protect their children from substance use is to confirm that they are an important member of the community:

[I] think … too many times kids don't feel … like they matter … The messages that tell them that they matter and that their … community values them.

It is important that any outside source that works with Abenaki people have cultural competency training, with an emphasis on AI/AN culture and history:

It's important that … the Vermont Department of Health or any other agency that's trying to work with the Abenaki people … have some general knowledge of the Abenaki, their culture … the historical trauma we all are part of.

Access to education and organizations that assist community members when they are dealing with substance abuse issues and the social systems that become involved after an individual asks for help were a primary concern:

grant funding, that's … a temporary thing … there's no … continuity … it's hard to build that trust when it's … piecemeal or not accessible.

Participants insisted that self-advocacy was a more effective solution than relying on outside institutions:

a lot of families that have a history of either mental illness or have dealt with it …. they … see the behavior … sometimes they don't know how to successfully address that if they haven't been taught.

One of the best solutions to addressing parenting and youth substance abuse issues is training and education to better understand the causes of substance abuse. Cultural competency training for outside organizations would illuminate the reasons why the Abenaki have hesitations when dealing with government institutions.

### Opioid use, prescription drug misuse, and the Abenaki community

While some Abenaki community members shared their efforts to avoid using opioids, others have found it hard to access other options to cope with physical and mental pain, as one participant noted,

opioid use is rampant in my generation. But at the same time, I've watched my wife go through crippling pain. I do believe opioids are a valid tool. But I also agree that you should not start the solution there.

Because opiates have the same stigma as other addictive substances, individuals battling physical pain turn to any substance that virtually has the same affects:

I have community members that have pain and they … don't take the opiates. They figure alcohol is a lesser evil.

Participants shared that opioids are often heavily prescribed as a first or second option for chronic pain relief and the plans to ween clients off of opioid dependency seem to be inadequate, “They prescribe it for way too long a period and then they see it's a problem.” Another challenge expressed by participants was that of poverty and socioeconomic challenges that turn opioids into a form a currency, as one person noted, “opioids … outside of the family, it's currency, it's tradeable.”

Participants also spoke at length about challenges with proper methods for disposal of prescription drugs, as they shared that it is part of Abenaki culture to not be wasteful, thus, culturally, many Abenaki struggle with retaining medications past expiration dates, as one person shared, “my mom … any medicine just stays in that cabinet, she doesn't need it anymore, I don't think she's ever returned to the drug store, to return it.” This sentiment was echoed by another participant who shared that, “properly disposed medications … I just wish there were more places than just police departments because a lot of people feel apprehensive about bringing narcotics into the police department.”

## Discussion and Health Equity Implications

Abenaki communities have a mistrust of formal education, law, health care, and mental health institutions due to institutionalized racism and oppression.^6,7^ Participants emphasized this point by sharing their apprehension and mistrust of traditional helping organizations. Health care professionals working with the Abenaki community need to be educated to understand the root causes of substance abuse and mental health disorders. The high prevalence of SUD and mental health in Indigenous communities is connected to historical trauma, structural racism, and loss and erasure of cultural identity, as participants within this Abenaki Indigenous community shared.^7,10^ Health, mental health, and wellness services need to be delivered by culturally competent providers who are invested in developing meaningful and trusting relationships with the Abenaki community to foster trust.

Educational, academic, and health care institutions should recruit members of the Abenaki into positions where they can advocate for their communities and specialize in treatments to reduce poverty, mental health disorders, substance abuse, and suicide rates. Finally, funding should be allocated to address behavioral health disparities across AI/AN communities, including but not exclusive to state recognized tribes, to ensure equitable access to resources and programs for historically underserved and unserved groups such as the Abenaki people.^13^

The Abenaki community will benefit from education on the harmful effects of prescription medicine use after expiration as this was a prominent theme within this study. Furthermore, various options for safe disposal should be explored. Participants shared that an incentive program may help members dispose of expired medication properly, as in a financial incentive if unused prescriptions are returned to a drop-off center at public events that serve the Abenaki community.

Fortunately, AI/AN individuals are seeking addiction and mental health disorder treatment options at higher rates, especially from traditional AI/AN wellness sources.^8,14^ Culturally relevant treatment should involve supporting the reclamation of cultural identity and spirituality, which have been shown to be efficacious in the treatment of substance use in AI/AN populations.^6,9^ Further research suggests that when AI/AN people embrace the positive characteristics of their culture and participate in traditional activities, especially those that strengthen family values and connection, incidences of substance use will decrease.^5,6,9,14^ Finally, substance use prevention, the promotion of physical and mental well-being, and reinforcing positive cultural identity strategies need to be introduced to AI/AN populations at a young age.

## Study Limitations and Recommendations for Future Work

This study had a number of limitations. This was a small qualitative study, and thus, generalizability was not possible. There was very little literature about the Abenaki, and thus, we had to look to more general statistics about AI/AN populations to inform this study. Those who participated likely had an interest and investment in this topic and including a larger and more diverse Abenaki sample is needed for future work.

Clearly, there is a need for much more research about how best to support Abenaki health and well-being, and this study is merely a starting point. More research with Abenaki communities is needed to better understand culturally relevant approaches to health and well-being for this population. Community-based participatory research methodologies hold promise for conducting ethical research with vulnerable populations such as the Abenaki.^15^

## Conclusion

There are many obstacles to effective prevention and intervention services such as limited funding, structural discrimination and subsequent mistrust of health institutions, lack of health care access, and a loss and erasure of Indigenous culture, as evidenced by the findings from this study with Abenaki community members.^16^ As many AI/AN persons seek treatment, it is imperative that AI/AN individuals are recruited into the health and mental health fields to address issues with discrimination and racism in health care.^13,16^ Many AI/AN individual's seek traditional holistic treatments and may be more willing to receive help from AI/AN practitioners, especially those who are knowledgeable in the lasting impacts of historical trauma and can provide high-quality mental health care, support, and services.

Addressing the need and prevalence of prescription drugs within this context merits further study. Finally, AI/AN culture and tradition in Vermont needs to be fostered in public educational and wellness organizations, as a mode of substance use and suicide prevention among Indigenous communities such as the Abenaki in Vermont.^17,18^

## Abbreviations Used

AI/AN: American Indian and Alaska Native
IRB: Institutional Review Board
NSDUH: National Survey on Drug Use and Health
SUD: substance use disorder
